# Major Depression as a Complicating Factor for Acute Coronary Syndrome

**DOI:** 10.5935/abc.20170122

**Published:** 2017-09

**Authors:** Levent Cerit

**Affiliations:** Near East University, Nicosia - Cyprus

**Keywords:** Acute Coronary Syndrome, Depression, Seorotin Uptake Inhibitors

Dear Editor,

I have read with great interest the article entitled "Major Depression and Acute Coronary Syndrome-Related Factors" by Figueiredo et al.,^1^ recently published in journal. The investigators found a 23% prevalence of patients with acute coronary syndrome (ACS) meeting the diagnostic criteria for major depressive disorder (MDD). Women were more susceptible to develop MDD in the sample group of ACS patients, with a three-and-a-half-time greater likelihood than men.^1^

Almost half of patients with ACS are affected by depression,^2^ many of them receiving antiplatelet therapy and often treated with a serotonin selective reuptake inhibitor (SSRI). Fluoxetine and fluvoxamine are potent inhibitors of CYP2C19.^3^ Clopidogrel is a pro-drug that undergoes a two-stage activation process, which is mediated by several cytochrome P450 (CYP) hepatic enzymes. CYP2C19 is involved in both activation steps, raising concern that the drug that inhibit CYP2C19 may decrease clopidogrel's effectiveness.^4^ Bykov et al.^5^ reported that treatment with a CYP2C19-inhibiting SSRI such as fluoxetine and fluvoxamine when initiating clopidogrel might be associated with slight decrease in its effectiveness.

In this context, considering not only the concomitant disorder with ACS but also the complicating factor due to CYP2C19-inhibiting SSRI treatment, MDD should be evaluated meticulously.
