# Supplementary material for: Major Depression as a Complicating Factor for Acute Coronary Syndrome
Source: Arq Bras Cardiol. 2017 Sep;109(3):271–2. doi: 10.5935/abc.20170122 (PMC5586238; doi:10.5935/abc.20170122)
Supplement: Related texts

# Reply

**Authors:** José Henrique Cunha Figueiredo, Nelson Albuquerque de Souza e Silva, Basilio de Bragança Pereira, Gláucia Maria Moraes de Oliveira

The authors are grateful for the comments made in a letter to the editor regarding the article Major Depression and Acute Coronary Syndrome-Related Factors, published in Arq Bras Cardiol. 2017; 108 (3): 217-227.

We agree with the relevant comments of the author of the letter, warning about the use of antidepressants in patients with acute coronary syndrome and their interaction with the antiplatelet Clopidogrel.

We complement the comments with the following considerations:

1) The effectiveness of Clopidogrel (a platelet P2Y12 receptor blocker), in its action as a platelet antiadhesive, among other known and unknown actions, is dependent on its conversion to an active metabolite by the cytochrome-dependent P450 (CYP- 450), mainly CYP2C19. Its effect as a platelet antiadhesive is lower in patients who are homozygous for non-functional alleles of CP2C19 genes, so-called "poor metabolizers". It is not recommended the use of Clopidogrel in these patients and, when possible, to identify these cases using genetic tests. Therefore, any other drug that interferes with the cytochrome dependent system altering the conversion of Clopidogrel to its active metabolite, mainly in the poor metabolizers, may reduce its effect.

Selective serotonin reuptake inhibitor antidepressants (such as fluoxetine, fluvoxamine, etc.) may interfere with the metabolism of clopidogrel, increasing the adverse effect of clopidogrel (by increasing the inhibition of the P2Y12 receptor) and, therefore, increasing the Risk of bleeding, decreasing the concentration of the active metabolite of Clopidogrel, mainly in the genetically "poor metabolizers", reducing its effectiveness.

2) Selective serotonin reuptake inhibitor antidepressants have multiple adverse cardiovascular effects such as: chest pain, angina pectoris, palpitation, arterial hypertension, prolonged *QT interval* on the *ECG*, ventricular arrhythmias, syndrome of inappropriate antidiuretic hormone secretion, increase of the risk of severe hyponatremias in patients already in use of diuretics, among innumerable adverse effects, which always more serious in patients with acute coronary syndrome.

3) Our article sought to initially verify the presence of the pattern (set of variables found in association) that is called "Major Depression" among patients with another pattern called Acute Coronary Syndrome and hospitalized in the hospital coronary units environment. In addition to this identification of variable patterns (diagnostics), we intend to study how and which, among the variables collected in these individuals, including sociodemographic variables, were associated. We do not intend to define causal associations, even because the design of the study would not allow such inferences. However, we intend to understand the interactions between variables present in a certain evolutionary state of patients' lives. This allows us a better clinical understanding of the person and, therefore, greater possibility of helping it during its evolution in this acute period and its subsequent evolution. We do not understand causality deterministically, that is, if the variable x is present then y will occur. We understand a person as a complex system that interacts with the environment in which he lives. Therefore, it is more important to understand the organization or interaction between component variables, which we can be identified in that complex system formed by people living in an environment (including the hospital environment), than to isolate possible "causal" variables, whether genetic or pharmacological and impute a direct causality that does not exist or that we only identify in simple or more stable systems. We believe that complex thinking is the basis of clinical judgment and that the important thing is to understand the biological system as a whole, due to its constant evolution or dynamics. For this, only observing the system in its continuous evolution.

We have chosen the linear log model to study these interactions as shown in Figures 1 and 2 of the article. The previous history of depression was one of the variables of that model. Certainly, these patients were already exposed to antidepressant drug use during their lifetime, but we cannot conclude, and it was not our goal, that these drugs "cause" coronary syndrome. However, from the clinical point of view, the associations found indicate that individuals and clinicians should be warned that antidepressants may be involved in the interactions of variables that make up the pattern Acute Coronary Syndrome and its complications (eg. arrhythmias ), and that this type of drug also interacts with other drugs such as antiplatelet drugs, diuretics and others, composing other patterns such as hyponatremia syndrome of inappropriate secretion of antidiuretic hormone, and even opposing patterns such as bleeding or loss of the efficacy of Clopidogrel.

Clinical thinking needs to detach itself from the bonds in which deterministic and causal thinking placed it and return to what has always been clinical judgment, now with a strong theoretical basis, complex thinking and complex adaptive dynamic systems. To do this we have to observe the evolution of the person throughout his life and his relationship or interaction with the environment in which he lives, including his social and cultural relations and understand the interactions between infinite variables always with a degree of uncertainty and considering the appearance of emerging or unpredictable phenomena.

**José Henrique Cunha Figueiredo** **Nelson Albuquerque de Souza e Silva** **Basilio de Bragança Pereira** **Gláucia Maria Moraes de Oliveira**
